# Overestimation of benefit when clinical trials stop early: a simulation study

**DOI:** 10.1186/s13063-022-06689-9

**Published:** 2022-09-05

**Authors:** Sharon Liu, Scott R. Garrison

**Affiliations:** 1 Faculty of Medicine & Dentistry, University of Toronto, Toronto, ON M5S 1A1 Canada; 2 Pragmatic Trials Collaborative, Faculty of Medicine & Dentistry, University of Alberta, 6-60 University Terrace, Edmonton, AB T6G 2T4 Canada

**Keywords:** Interim analysis, Truncated trials, Stopping early, Overestimation, Winner’s curse, Selection bias, Monte Carlo methods

## Abstract

**Background:**

Stopping trials early because of a favourable interim analysis can exaggerate benefit. This study simulated trials typical of those stopping early for benefit in the real world and estimated the degree to which early stopping likely overestimates benefit.

**Methods:**

From 1 million simulated trials, we selected those trials that exceeded interim stopping criteria, and compared apparent benefit when stopped with the true benefit used to generate the data. Each simulation randomly assigned period of observation, number of subjects, and control event rate using normal distributions centred on the same parameters in a template trial typical of real-world “truncated” (i.e. stopped for benefit) trials. The intervention’s true relative risk reduction (RRR) was also randomized, and assumed 1% of drugs have a warfarin-like effect (60% RRR), 5% a statin-like effect (35% RRR), 39% an ASA-like effect (15% RRR), 50% no effect (0% RRR), and that 5% would cause harm (modelled as a 20% relative risk increase). Trials had a single interim analysis and a *z*-value for stopping of 2.782 (O’Brien-Fleming threshold). We also modelled (1) a large truncated trial based on the SPRINT blood pressure trial (using SPRINT’s parameters and stopping criteria) and (2) the same typical truncated trials if they instead went to completion as planned with no interim analysis.

**Results:**

For typical truncated trials, the true RRR was roughly 2/3 the observed RRR at the time of stopping. RRR was overestimated by an absolute 14.9% (median, IQR 6.4–24.6) in typical truncated trials, by 5.3% (IQR −0.1 to 11.4) in the same trials if instead carried to completion, and by 2.3% (IQR 0.98–1.09) in large SPRINT-like trials. For all models, to keep the absolute RRR overestimate below 5%, 250 events were required.

**Conclusion:**

Simulated trials typical of those stopping early for benefit overestimate the true relative risk reduction by roughly 50% (i.e. the true RRR was 2/3 of the observed value). Overestimation was much smaller, and likely unimportant, when simulating large SPRINT-like trials stopping early. Whether trials were large or small, stopped early or not, a minimum 250 events were needed to avoid overestimating relative risk reduction by an absolute 5% or more.

**Supplementary Information:**

The online version contains supplementary material available at 10.1186/s13063-022-06689-9.

## Introduction

When we predicate our attention, or action, on a clinical trial demonstrating statistically significant benefit, we set ourselves up, on average, to overestimate the benefit of that intervention. This is because trials that randomly deviate towards greater benefit than is reality are more likely to meet the benefit threshold that we set. This form of bias has been referred to as the “winner’s curse” [1]. Its effects are strongest when only large differences can be declared statistically significant, and this makes it potentially problematic when deciding whether or not to stop a trial early for benefit based on interim data.

Interim analyses have a large effect, both positive and negative, on the practice of medicine. An early look at the data can accelerate patient access to life-saving therapies and reduce the cost of developing drugs with little promise. On the other hand, when trials stop early for benefit, they are more likely to influence clinical practice, and this can have a chilling effect on further research [2–4]. It could be years before such trials are reproduced, and when trials are “truncated” (stopped early for benefit), we lose many patient-years of observation that would have helped us to understand potential harms. Truncated trials are also problematic for health care regulators and funders, who may use the trial’s point estimate of effect in decisions surrounding approval, access, and reimbursement, with little consideration of the degree to which early stopping may have biased the effect estimate. Given the outsized influence of truncated trials, it is clinically important to understand the degree to which such trials are likely to overestimate benefit. In this study, we use computer simulation of typical truncated trials to quantify their likely overestimation of benefit in the real world, and to determine the number of events that need to be observed to ensure reasonably accurate effect estimates.

## Methods

In 2005, Montori et al. published a systematic review identifying randomized trials stopped early for benefit [5]. We built 1 million trial simulations around a template truncated-trial typical of those identified in the Montori review, selected out those trial iterations which exceeded their stopping criteria at interim analysis, and compared the apparent benefit at the time these trials would have stopped, with the true benefit used to generate the data. All modelling and analyses were carried out using R software version 4.0.3.

### Simulation of trial parameters

The template trial we built our model on was a comparison of low-intensity warfarin versus placebo for the prevention of recurrent venous thromboembolism [6]. We chose this trial because it was highly typical of the truncated trials identified in the Montori et al. systematic review so far as mean period of observation at the time of early stopping (2.1 years), total number of subjects randomized 1:1 into intervention or control (508), and control group event rate (7.2 events per 100 person-years). For each simulated trial, we randomly assigned these three characteristics using normal distributions centred on the template trial’s values, and having standard deviations (STD) 25% of the mean. A 25% STD ensured 95% of these simulated trial characteristics were within ± 50% of the template value. The Montori et al. systematic review identified truncated trials as most commonly having a single interim analysis, and utilizing O’Brien-Fleming stopping rules. Hence, our simulation used a *z*-value for stopping of 2.782 since this was the O’Brien-Fleming boundary point for a single interim analysis with 50% of study information available [7]. To investigate the effect of the stopping boundary on overestimation we further examined the degree of overestimation over a range of z-values from 2.0 to 4.0, with steps of 0.25.

### Simulation of “true” benefit

The degree of true benefit was randomly assigned and expressed as a relative risk reduction (RRR). To determine this, we sought consensus from 4 physicians and 4 pharmacists, each an experienced reviewer of drugs targeted at primary care providers, as to their expectations of true benefit for first-in-class phase-3 trials where the intervention is targeted at preventing adverse events (e.g. heart attack or stroke) in those at risk because of common conditions such as diabetes, hypertension, or atrial fibrillation. Following discussion, the group’s consensus estimate was that 1% of drugs would have a warfarin-like effect (60% RRR), 5% a statin-like effect (35% RRR), 39% an ASA-like effect (15% RRR), 50% no effect (0% RRR), and that 5% would cause harm (modelled as a 20% relative risk increase). Simulated trials were first randomized to one of these 5 basic categories using the consensus probabilities, and then assigned a specific RRR (used for all simulated subjects in that trial) using a normal distribution centred on the category benefit with a 15% STD. The resulting distribution for assigned true benefit for the trials in our simulations visually resembles a normal distribution centred on a 7% RRR (see Additional file 1, Fig. S1) and having a range of benefit somewhat wider than that described by the consensus categories due to our using normal distributions to assign the values. To investigate whether our true benefit assumptions materially influenced our findings, we carried out the same analysis using both more generous assumptions of benefit (2% warfarin-like, 8% statin-like, 50% ASA-like, 40% no effect, no drugs harmful) and less generous assumptions of benefit (0.5% warfarin-like, 2.5% statin-like, 29% ASA-like, 60% no effect, 8% harmful).

### Simulation of trial designs that would have higher power

For broader perspective, we also simulated:

- The same *typical truncated trials if they instead went to their planned completion* with no interim analysis—for which we employed *z* >1.96, and centred our probability distributions on 750 subjects and 4 years observation (our template trial’s powered for parameters).
- A *large truncated trial*—for which we used the SPRINT blood pressure target trial as our template (period of observation 3.26 years, total subjects 9361, control group event rate 2.19 events per 100 person-years, and *z-*value when stopped 2.358).

## Results

Table 1 shows the characteristics of all simulated trials achieving statistical significance, either at interim analysis (for “typical truncated trials” and “large truncated trials”), or at trial completion (for “typical truncated trials if carried to completion”). For comparison, the online supporting material provides characteristics of all simulated trials, not just those meeting the criteria for statistical significance (Additional file 1: Table S1). Truncation occurred in 4.7% (47,174/1,000,000) of “typical” trial simulations, and 29.6% (295,602/1,000,000) of “large” trial simulations.

**Table 1:** Characteristics of simulated trials observing statistically significant benefit

Characteristic	Typical truncated trials (***n***=47,174)	Typical truncated trials if carried to completion (***n***=242,829)	Large truncated trials (***n***=295,602)
Number of participants	541 (456–626)	771 (648–896)	9,616 (8070–11,160)
Average follow-up (months)	27.1 (23.0–31.2)	49.9 (42.0–57.9)	40.2 (33.8–46.7)
Placebo event rate (per 100 person-years)	8.6 (7.3–10.0)	7.7 (6.5–9.0)	2.3 (1.9–2.7)
*Z*-value for stopping	2.782	1.960	2.358
Number of events	72 (53–96)	194 (142–259)	588 (429–784)
Number of trials that overestimate benefit	42,191 (89.4)	181,218 (74.6)	195,854 (66.3)
Number of trials that underestimate benefit	4983 (10.6)	61,611 (25.4)	99,748 (33.7)
True RRR (%)	38.6 (27.9–50.9)	27.0 (18.7–36.6)	26.0 (18.9–35.0)
Observed RRR (%)	54.8 (47.5–63.6)	32.3 (25.6–41.2)	28.1 (21.9–36.7)
Absolute RRR overestimate (%)	14.9 (6.4–24.6)	5.3 (−0.1 to 11.4)	2.3 (−1.3–6.3)
Observed RRR/true RRR	1.37 (1.12–1.83)	1.18 (0.99–1.51)	1.08 (0.96–1.28)
Number of trials with negative true benefit (i.e. harm)	265 (0.6)	2172 (0.9)	456 (0.2)
Number of trials with observed RRR/true RRR in range			
1.0–1.2	10,559 (22.4)	63,803 (26.3)	99,369 (33.6)
1.2–1.5	12,194 (25.8)	53,165 (21.9)	56,779 (19.2)
> 1.5	19,173 (40.6)	62,078 (25.6)	39,250 (13.3)

Data are median (IQR), or number (%)

For “Typical truncated trials” and “Large truncated trials”, this refers to statistical significance at the time of interim analysis, when an early stopping decision is being made. For “Typical truncated trials if carried to completion”, this refers to statistical significance at trial conclusion if no interim analysis is carried out

**Fig. 1:**
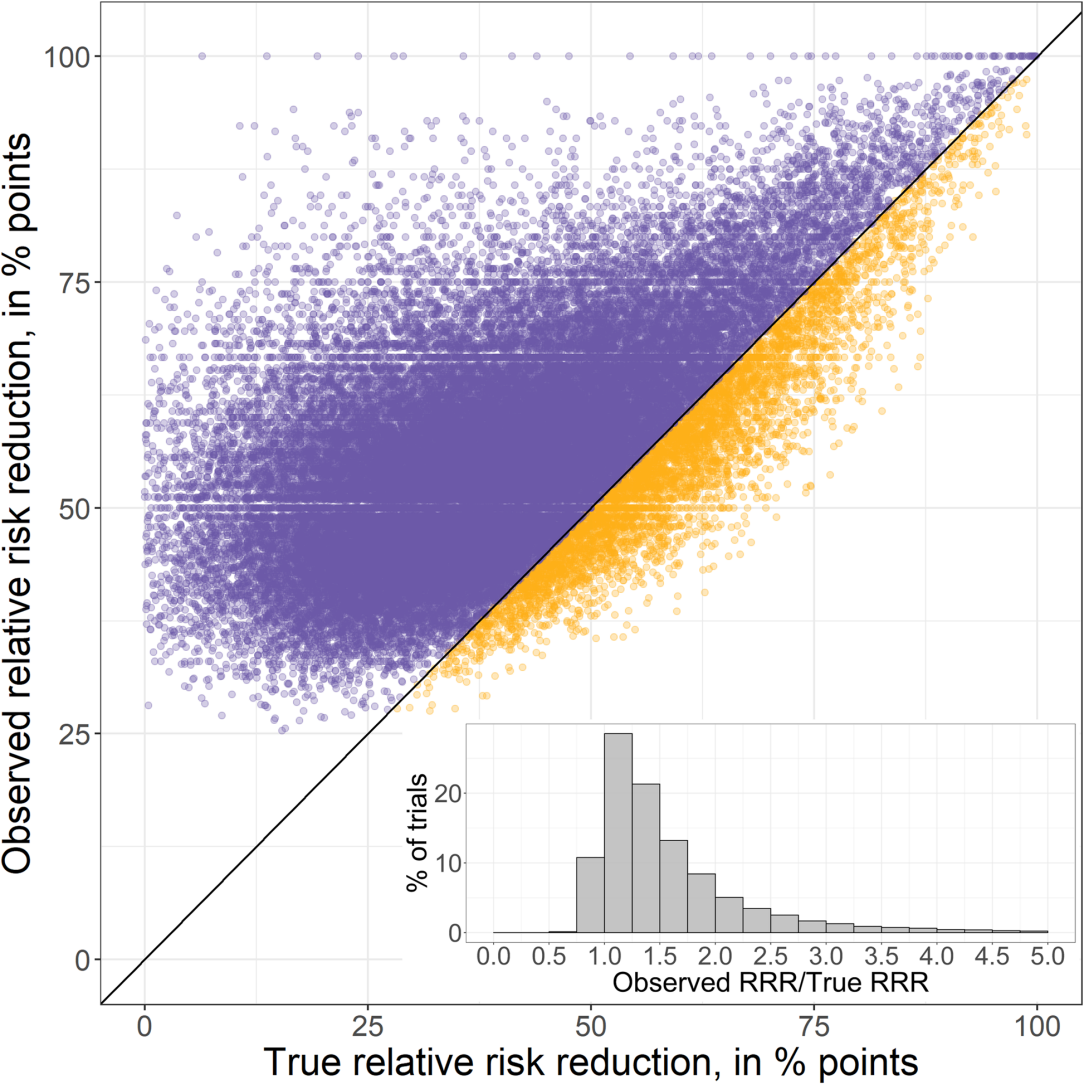
Scatterplot of observed relative risk reduction versus true relative risk reduction for all simulated typical truncated trials where true benefit existed. Figure shows overestimates in purple and underestimates in orange. Overestimates are 8.5 times more numerous than underestimates. Superimposed is a histogram of observed RRR/true RRR to show the range of overestimation

Our simulated “typical truncated trials” had (1) a median 72 (IQR 53–96) events at stopping, as compared to 66 (IQR 23–195) for the real-world truncated trials identified by the Montori systematic review, and (2) a median observed RRR of 54.8% (IQR 47.5–63.6%), as compared to a median 47% RRR (IQR 34–72%) in the Montori identified trials. For the main analysis of “typical truncated trials”, we show a scatterplot of observed relative risk reduction versus true relative risk reduction at the time of interim analysis for all simulated trials where a true benefit existed (Fig. 1). The observed relative risk reduction exceeded the true benefit 8.5 times more often than it fell below it. These “typical truncated trials” collectively overestimate the true RRR by a median absolute 14.9% (IQR 6.4–24.6%) with a ratio of observed RRR/true RRR of 1.37 (IQR 1.12–1.83). Assigning a greater degree of true benefit to the intervention (Additional file 1: Table S2) provided near identical results, with a median absolute overestimate of the true RRR of 15.2% (IQR 7.5–23.9) and an observed RRR/true RRR of 1.31 (IQR 1.14–1.57). On the other hand, assigning lesser true benefit to the intervention led to substantially greater overestimation of benefit (Additional file 1: Table S3), with a median absolute overestimate of the true RRR of 23.9% (IQR 12.7–35.9) and an observed RRR/true RRR of 1.64 (1.24–2.35).

When varying the *z*-threshold (Additional file 2: Table S2), we found the degree of overestimation to be largely independent of the stopping threshold when *z* was between 2.0 and 3.0 (15.7 to 14.5%). With larger *z*-values than this, the degree of overestimation gradually became smaller, but was still 11.1% at a *z* of 4.0. Although overestimates were common, it was rare to find statistically significant benefit when the true RRR was negative (i.e. when the intervention was harmful). This occurred in fewer than 1% of all 3 simulated trial types.

**Fig. 2:**
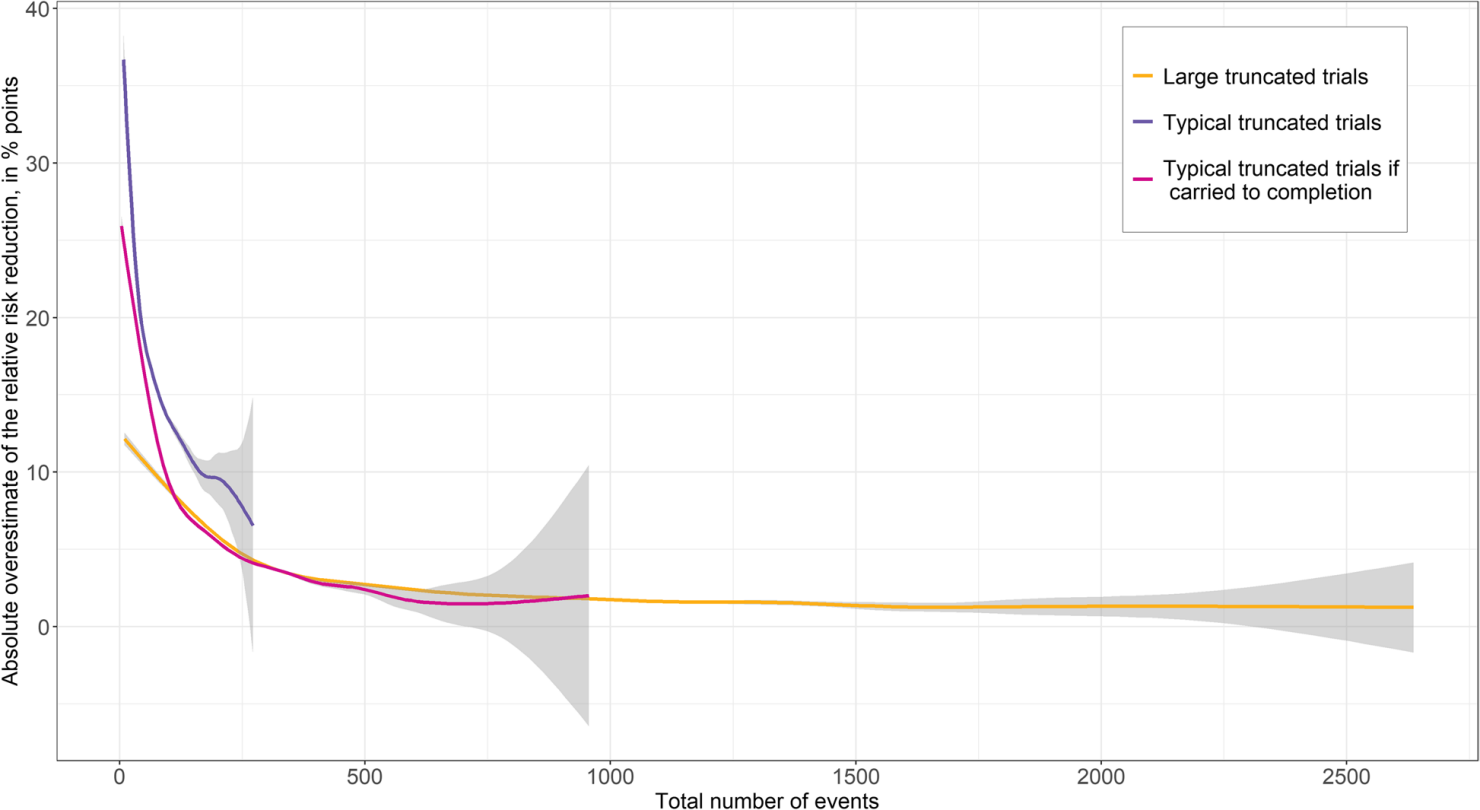
Absolute overestimate of the relative risk reduction versus the total number of outcome events in simulated trials. Coloured trendlines, along with grey-shaded areas representing the 95% confidence intervals, are created using a generalized additive model

**Fig. 3:**
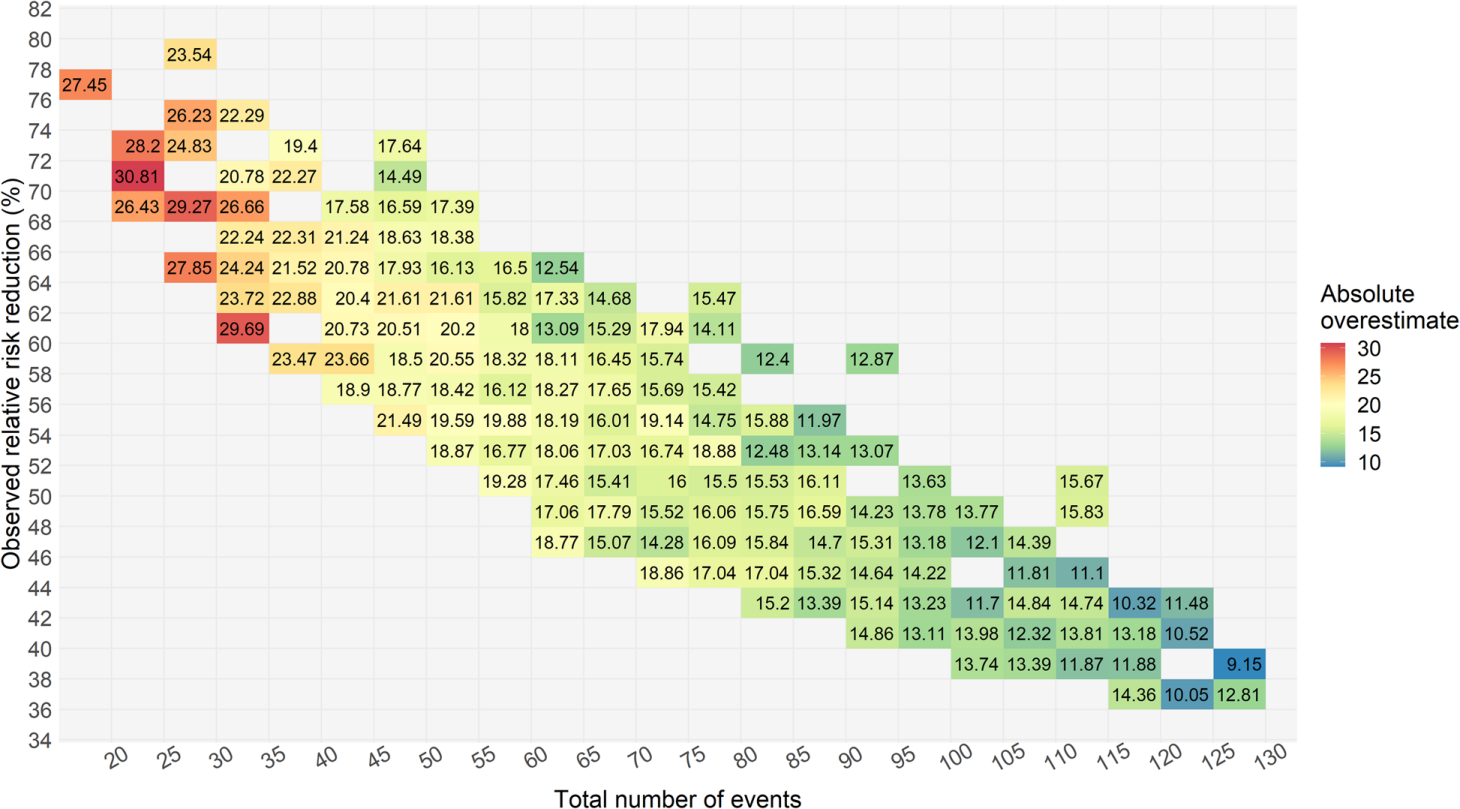
Absolute overestimate of the relative risk reduction as a function of the number of observed events and the observed relative risk reduction in simulated typical truncated trials. Data are a “heatmap” of the absolute overestimate of the relative risk reduction showing all cells for which there were at least 50 simulated trials. The range over which data is displayed is the range over which our model predictions can be applied.

The number of events was the main determinant of the degree of RRR overestimate (Fig. 2), but there was also a relationship with observed RRR such that the overestimate was highest at an observed RRR of 67% (Additional file 1: Fig. S2). Both of these figures display our absolute RRR overestimates for all trials reaching statistical significance as a best-fit trendline and 95% confidence interval (versus number of events in Fig. 2, or observed RRR in Fig. S2), as created using the generalized additive model algorithm (with restricted maximum likelihood method) available in R software’s ggplot package. A heatmap of the absolute RRR overestimate, versus event number and observed RRR, is shown in Fig. 3 and provides an estimate of the absolute RRR overestimate for every cell with a minimum of 50 simulated trials.

The absolute RRR overestimates were much lower in “typical truncated trials if carried to completion” (median 5.3% IQR −0.1 to 11.4%) and “large truncated trials” (median 2.3% IQR −1.3 to 6.3%). When each of these three trial types are plotted together to examine absolute RRR overestimate versus the number of events (Fig. 2), we see that these curves are largely asymptotic over much of their range. This is consistent with the number of events being the main determinant of the RRR overestimate regardless of the trial type (large or small, stopped early or not). For a drug treating a common condition, where widespread use might be expected, we would consider a ≥ 5% absolute overestimate of the relative risk reduction to be clinically important. Using Fig. 2, we see that the RRR overestimate is below an absolute 5%, when approximately 250 or more events have been observed.

## Discussion

Using Monte Carlo simulation, we estimate trials typical of those being stopped early for benefit to overestimate the relative risk reduction, on average, by an absolute 14.9%—an amount comparable to the established benefit of many therapeutics. Overestimates are much smaller when such trials are taken to completion without interim analysis (5.3%) and when large SPRINT-like trials are stopped early (2.3%). Although the magnitude of benefit is frequently, and substantially, overestimated in typical truncated trials, in fewer than 1% of such trials was the intervention actually harmful. Hence, while errors of magnitude (“type M” errors) were common, errors in the direction of effect (“type S” errors) were rare [8].

**Fig. 4:**
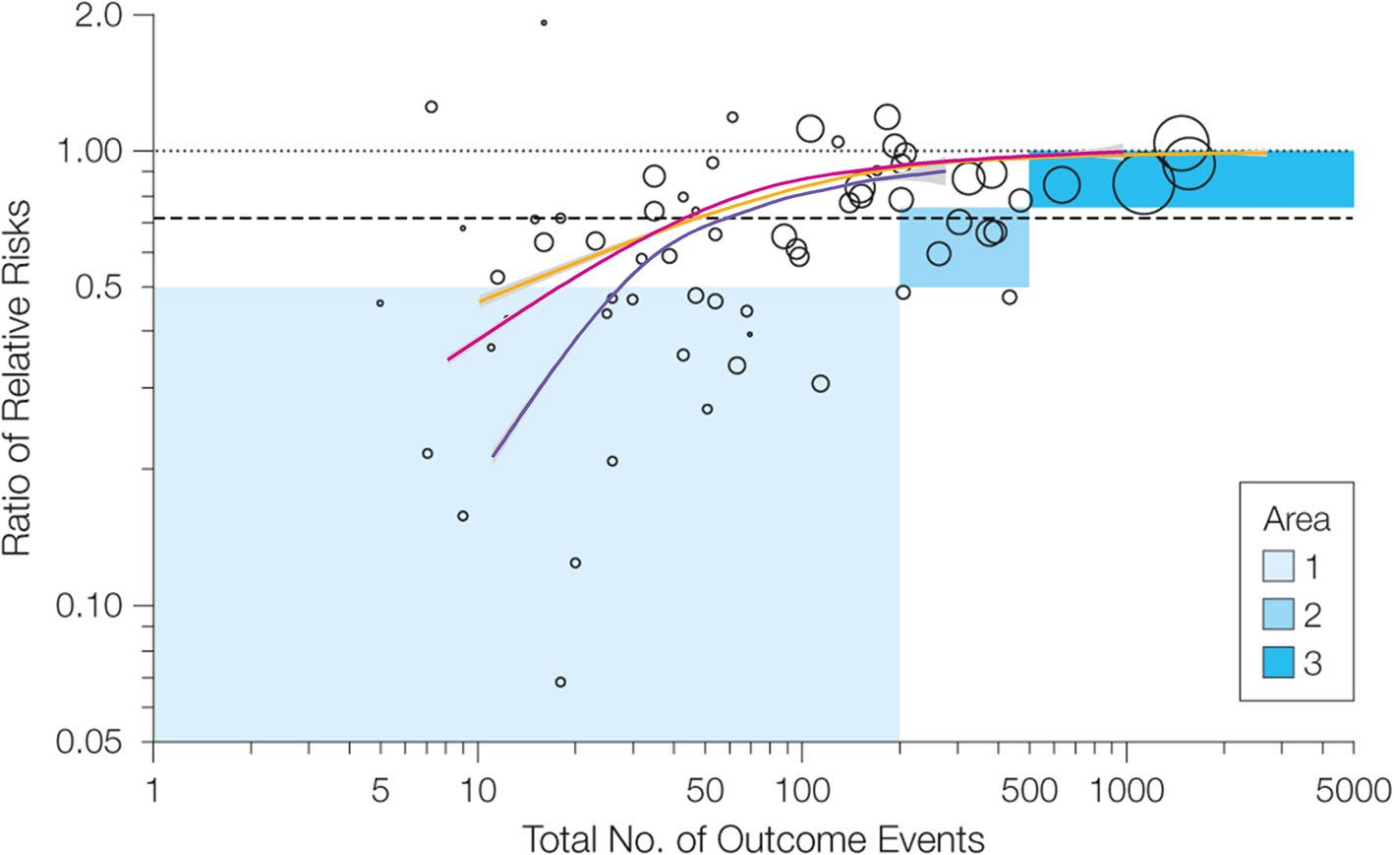
Comparison of our model’s predictions for overestimation of benefit with real-world overestimates of effect. Figure shows our model predictions, displayed as observed relative risk reduction/true relative risk reduction, overlayed on top of the summary figure from Bassler et al. [9]*.* The Bassler estimates come from comparing the effect estimate in truncated trials with non-truncated trials answering the same question. As in our Figure 2, the purple line represents typical truncated trials, the red line represents typical truncated trials if instead carried to completion, and the orange line represents large truncated trials

Our simulation is consistent with a real-world examination of 91 truncated trials stopped early for benefit [9]. When compared to matched non-truncated trials answering the same question, these real-world truncated trials showed apparent overestimates of benefit that align closely to those our model predicts (Fig. 4). Our findings are also consistent with the work of Pocock and Hughes, who have previously used clinical trial simulation to demonstrate that interim analysis stopping criteria tend to exaggerate the treatment effect [10], and with the work of Walter et al. who present mathematical models for the relative overestimates that could be expected for small, medium, and large trials [11].

Our simulation is limited by the need to make assumptions about the likelihood and magnitude of the intervention’s benefit and is valid only over the range of observed benefit, and number of observed events, displayed in our heatmap. Vaccines, for instance, have a much higher expectation of benefit (perhaps 70% RRR or more) and would need to be simulated separately. Our simulation is also limited by the absence of meaningful covariates in the analysis. This might explain the slightly smaller RRR at the time of stopping in the Montori identified truncated trials, compared to our simulated trials. This smaller RRR could also be explained by less restrictive stopping criteria in some real-world trials. Of the 143 trials Montori identified, 28 (20%) were not applying a higher statistical stopping threshold during their interim analysis, and another 28 trials either did not describe their monitoring methods, or did not state a prespecified *p*-value for stopping.

We have also made the simplifying assumption that trial characteristics can vary independently. In reality, control event rate, number of subjects, and length of follow-up are interdependent when trials are being designed. Modelling them as independent may have produced some simulated trials with a power too low for the trial to be pursued in the real world. However, our conclusions derive only from those simulated trials which do demonstrate significant benefit. Our analysis of trial simulations is simultaneously strengthened by choosing a template trial that systematic review suggests to be typical of truncated trials, by the ability to know the true benefit used to generate the data, and by avoiding real-world confounding such as publication bias, unequal distribution of risk factors, and a host of methodologic biases that could be introduced through study conduct or design. Although our assumptions for true benefit came from a panel of drug review experts (i.e. were opinion based), assuming greater benefit yielded near identical results, while assuming lesser benefit yielded substantially greater overestimates, suggesting our findings may be conservative.

## Conclusion

The statistical hurdle we impose on trials before they warrant our attention, or our action, introduces a selection bias that leads us to overestimate benefit. The extent of that overestimation varies nonlinearly with the number of events observed and diminishes only slightly with stricter stopping criteria. To keep from overestimating the relative risk reduction by more than an absolute 5%, trials of common primary care implementable interventions targeted at cardiovascular risk reduction (whether large or small, stopping early or not) may wish to continue until a minimum of 250 events have been observed. A “long-shot” intervention, with a lower initial likelihood of benefit, would require even more events for confidence in the magnitude of effect. Researchers in other therapeutic areas (e.g. oncology) may wish to avoid overestimation of benefit by employing analogous trial simulations, and establishing their own therapeutic area-specific minimum event thresholds.

## Supplementary Information

**Additional file 1: Figure S1.** Histogram of the true relative risk reduction assumed in simulated trials. **Table S1.** Characteristics of all simulated trials at time significance was assessed. **Table S2.** Characteristics of simulated trials observing statistically significant benefit if there is GREATER expectation of benefit. **Table S3.** Characteristics of simulated trials observing statistically significant benefit if there is LESS expectation of benefit. **Figure S2.** Absolute overestimate of the relative risk reduction versus observed relative risk reduction in simulated trials.

**Additional file 2: Table S2.** Characteristics of simulated typical truncated trials observing significant benefit when z is varied.

## Data Availability

Data files with the parameters describing all 1 million trials created for each of the three model simulations, and a file containing the R programming code used to create the simulations, is freely available for download at www.PragmaticTrials.ca.
