# Supplementary material for: Overestimation of benefit when clinical trials stop early: a simulation study
Source: Trials. 2022 Sep 5;23:747. doi: 10.1186/s13063-022-06689-9 (PMC9446780; doi:10.1186/s13063-022-06689-9)
Supplement: Supplementary file 1 — Additional file 1: Figure S1. Histogram of the true relative risk reduction assumed in simulated trials. Table S1. Characteristics of all simulated trials at time significance was assessed. Table S2. Characteristics of simulated trials observing statistically significant benefit if there is GREATER expectation of benefit. Table S3. Characteristics of simulated trials observing statistically significant benefit if there is LESS expectation of benefit. Figure S2. Absolute overestimate of the relative risk reduction versus observed relative risk reduction in simulated trials. [file 13063_2022_6689_MOESM1_ESM.pdf]

Supporting Information

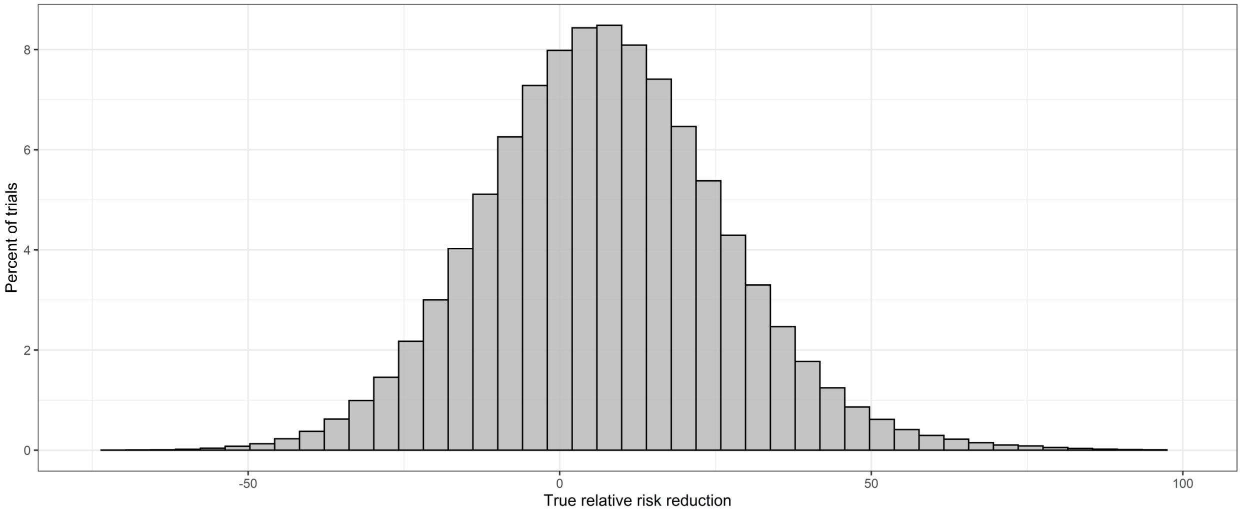

Fig. S1. Histogram of the true relative risk reduction assumed in simulated trials**.** This figure shows the end result of our process for assigning true benefit to simulated trial and results from assuming 1% of drugs would have a warfarin-like effect (60% RRR), 5% a statin-like effect (35% RRR), 39% an ASA-like effect (15% RRR), 50% no effect (0% RRR), and that 5% would cause harm (modelled as a 20% relative risk increase). Simulated trials were first randomized to one of these 5 basic categories using the consensus probabilities, and then assigned a specific RRR (used for all simulated subjects in that trial) using a normal distribution centered on the category benefit with a 15% STD. The resulting distribution for assigning true RRR resembles a normal distribution centered on a 7% RRR.

Table S1. Characteristics of all simulated trials at time significance was assessed

**Characteristic**	**Typical truncated trials (n = 1 million)**	**Typical truncated trials if carried to completion (n = 1 million)**	**Large truncated trials (n = 1 million)**
Number of participants	508 (423-594)	750 (623-876)	9,358 (7,781-10,936)
Average follow-up (months)	25.2 (21.0-29.5)	48.0(39.9-56.1)	39.1 (32.5-45.7)
Placebo event rate (per 100 person-years)	7.1 (5.7-8.6)	7.2 (5.9-8.5)	2.2 (1.8-2.6)
Z-value at assessment	2.782	1.960	2.358
Number of events	69 (49-94)	194 (138-262)	600 (428-812)
Number of trials that overestimate benefit	500,733 (50.1)	500,600 (50.1)	499,907 (50.0)
Number of trials that underestimate benefit	499,168 (49.9)	499,375 (49.9)	500,085 (50.0)
True RRR (%)	6.8 (-5.7-19.5)	6.7 (-5.7-19.5)	6.8 (-5.7-19.5)
Observed RRR (%)	7.4 (-13.2-25.8)	7.0 (-8.5-21.9)	6.9 (-6.9-20.6)
Absolute RRR overestimate (%)	0.0 (-14.7-12.8)	0.0 (-7.8-7.2)	0.0 (-5.0-4.7)
Observed RRR / True RRR	1.00 (0.86-1.16)	1.00 (0.92-1.09)	1.00 (0.95-1.06)
Number of trials with negative true benefit (i.e. harm)	357,300 (35.7)	357,977 (35.8)	357,564 (35.8)
Number of trials with observed RRR / True RRR in range			
1.0 – 1.2	70,177 (7.0)	118,991 (11.9)	167,335 (16.7)
1.2 – 1.5	85,660 (8.6)	114,977 (11.5)	126,714 (12.7)
> 1.5	345,306 (34.5)	267,055 (26.7)	206,874 (20.7)

Data are median (IQR), or number (%)

Table S2. Characteristics of simulated trials observing statistically significant benefit if there is GREATER expectation of benefit

Characteristic	Typical truncated trials<br><br>(n = 144,834)	Typical truncated trials if carried to completion<br>(n = 539,524)	Large truncated trials<br><br>(n = 817,915)
Number of participants	508 (432-586)	489 (411-567)	9,215 (7,708-10,732)
Average follow-up (months)	25.1 (21.4-28.9)	47.8 (40.3-55.4)	37.9 (31.7-44.1)
Placebo event rate (per 100 person-years)	7.0 (5.9-8.1)	6.2 (5.2-7.2)	2.1 (1.8-2.5)
Z-value at assessment	2.782	1.960	2.358
Number of events	48 (36-63)	86 (63-115)	465 (337-622)
Number of trials that overestimate benefit	132,966 (91.8)	387,532 (71.8)	457,995 (56.0)
Number of trials that underestimate benefit	11,868 (8.2)	151,992 (28.2)	359,920 (44.0)
True RRR (%)	48.5 (39.9-57.2)	41.9 (33.4-50.6)	38.3 (29.9-47.3)
Observed RRR (%)	64.0 (56.9-71.2)	47.2 (39.0-56.5)	38.9 (30.3-48.4)
Absolute RRR overestimate (%)	15.2 (7.5-23.9)	5.6 (-0.9-12.6)	0.8 (-2.8-4.5)
Observed RRR / True RRR	1.31 (1.14-1.57)	1.13 (0.98-1.34)	1.02 (0.93-1.12)
Number of trials with negative true benefit (i.e. harm)	8 (< 0.1)	125 (< 0.1)	32 (< 0.1)
Number of trials with observed RRR / True RRR in range			
1.0 – 1.2	37,919 (26.2)	172,124 (31.9)	337,645 (41.3)
1.2 – 1.5	51,301 (35.4)	135,482 (25.1)	96,300 (11.8)
> 1.5	43,738 (30.2)	79,801 (14.8)	24,018 (2.9)

Data are median (IQR), or number (%)

Table S3. Characteristics of simulated trials observing statistically significant benefit if there is LESS expectation of benefit

**Characteristic**	**Typical truncated trials**<br><br>**(n = 21,752)**	**Typical truncated trials if carried to completion**<br>**(n = 126,817)**	**Large truncated trials**<br><br>**(n = 228,307)**
Number of participants	505 (429-585)	492 (413-570)	9,378 (7,889-10,882)
Average follow-up (months)	25.0 (21.2-28.8)	48.2 (40.6-55.8)	38.6 (32.4-44.8)
Placebo event rate (per 100 person-years)	7.3 (6.1-8.5)	6.5 (5.4-7.5)	2.2 (1.8-2.6)
Z-value at assessment	2.782	1.960	2.358
Number of events	50 (37-66)	96 (70-128)	532 (389-707)
Number of trials that overestimate benefit	20,480 (94.2)	110,605 (87.2)	158,649 (69.5)
Number of trials that underestimate benefit	1,272 (5.8)	16,212 (12.8)	69,658 (30.5)
True RRR (%)	35.7 (23.9-49.7)	26.5 (17.2-36.7)	25.0 (18.2-33.3)
Observed RRR (%)	61.5 (54.1-70.4)	39.6 (32.8-48.3)	27.8 (22.1-35.7)
Absolute RRR overestimate (%)	23.9 (12.7-35.9)	12.7 (4.9-21.6)	2.9 (-0.9-7.2)
Observed RRR / True RRR	1.64 (1.24-2.35)	1.43 (1.11-2.05)	1.11 (0.97-1.34)
Number of trials with negative true benefit (i.e. harm)	386 (1.8)	3,363 (2.7)	581 (0.3)
Number of trials with observed RRR / True RRR in range			
1.0 – 1.2	3,224 (14.8)	22,096 (17.4)	72,229 (31.6)
1.2 – 1.5	4,342 (20.0)	27,297 (21.5)	47,609 (20.9)
> 1.5	12,528 (57.6)	57,849 (45.6)	38,230 (16.7)

Data are median (IQR), or number (%)

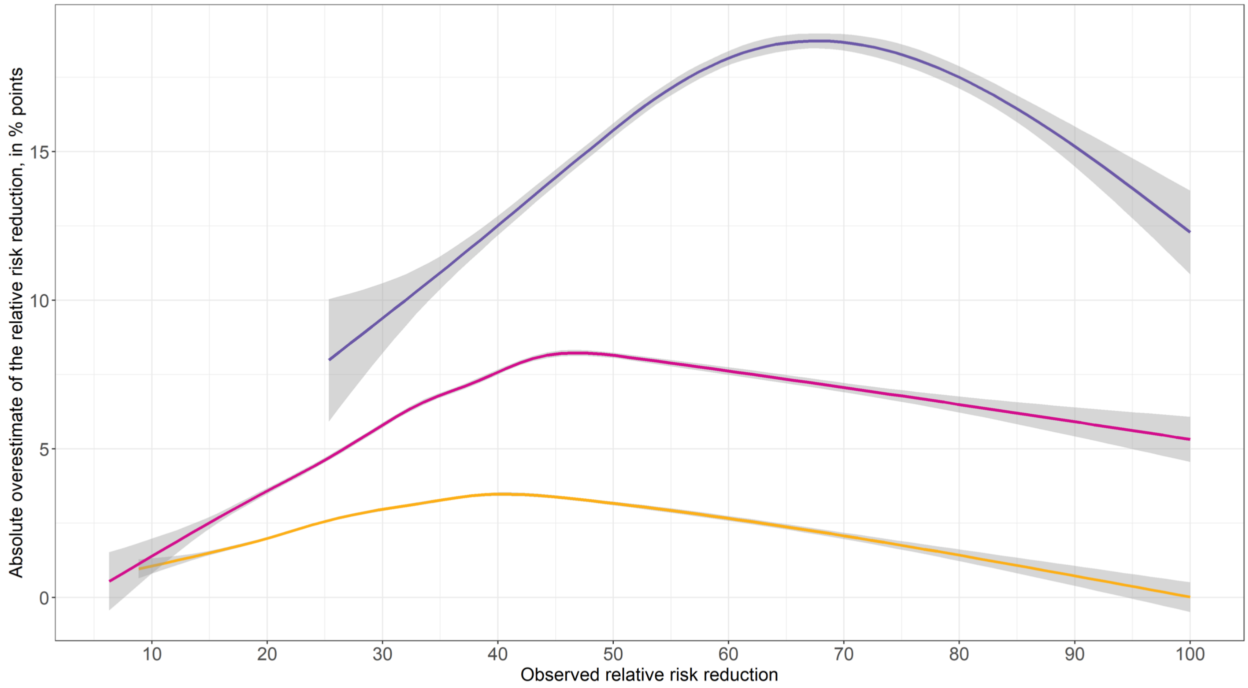

Fig. S2. Absolute overestimate of the relative risk reduction versus observed relative risk reduction in simulated trials. Coloured trendlines, along with grey shaded areas representing the 95% confidence intervals, are created using a generalized additive model.
