# Supplementary material for: Overestimation of benefit when clinical trials stop early: a simulation study
Source: Trials. 2022 Sep 5;23:747. doi: 10.1186/s13063-022-06689-9 (PMC9446780; doi:10.1186/s13063-022-06689-9)
Supplement: Supplementary file 2 — Additional file 2: Table S2. Characteristics of simulated typical truncated trials observing significant benefit when z is varied. [file 13063_2022_6689_MOESM2_ESM.pdf]

Table S2. Characteristics of simulated typical truncated trials observing significant benefit when z is varied

Characteristic	Z=2	Z=2.25	Z=2.5	Z=2.75	Z=3.0	Z=3.25	Z=3.5	Z=3.75	Z=4.0
# trials with significant benefit	120810	90869	67261	49144	35787	25783	17241	13268	9436
Number participants	528 (444-612)	531 (448-616)	536 (453-620)	541 (459-625)	546 (464-629)	550 (469-633)	556 (475-638)	559 (479-641)	564 (485-645)
Average follow-up (months)	26.3 (22.2-30.5)	26.5 (22.4-30.7)	26.8 (22.7-30.9)	27.1 (23.0-31.2)	27.3 (23.2-31.4)	27.6 (23.5-31.6)	27.9 (23.8-31.9)	28.0 (24.0-32.0)	28.4 (24.3-32.3)
Placebo event rate / 100 person-years	8.3 (7.0-9.7)	8.4 (7.1-9.8)	8.5 (7.2-9.9)	8.6 (7.3-10.0)	8.7 (7.3-10.1)	8.8 (7.4-10.2)	8.9 (7.5-10.2)	8.9 (7.6-10.3)	9.0 (7.7-10.3)
Total event number	70 (50-93)	70 (51-94)	71 (52-94)	72 (53-96)	73 (54-97)	74 (55-98)	75 (56-98)	75 (56-98)	76 (57-99)
Placebo events	46 (34-59)	47 (35-61)	48 (37-62)	50 (38-64)	51 (40-65)	53 (41-67)	55 (43-69)	56 (44-70)	57 (45-71)
Intervention events	24 (16-34)	23 (15-33)	23 (15-33)	22 (14-32)	22 (14-32)	21 (13-31)	20 (12-30)	19 (12-29)	19 (11-28)
# trials overestimating benefit	107721 (89.2)	81376 (89.6)	60292 (89.6)	43968 (89.5)	31945 (89.3)	22940 (89.0)	15288 (88.7)	11707 (88.2)	8274 (87.7)
# trials underestimating benefit	13089 (10.8)	9493 (10.4)	6969 (10.4)	5176 (10.5)	3842 (10.7)	2843 (11.0)	1953 (11.3)	1561 (11.8)	1162 12.3)
True RRR (%)	30.0 (19.5-41.5)	32.6 (22.0-44.3)	35.4 (24.7-47.3)	38.3 (27.5-50.5)	41.2 (30.3-53.7)	44.4 (33.3-57.1)	48.4 (36.9-61.2)	51.2 (39.8-63.8)	54.7 (43.3-67.0)
Observed RRR (%)	46.3 (39.0-55.6)	49.0 (41.9-58.1)	51.9 (44.6-60.7)	54.43 (47.2-63.3)	56.9 (50.0-65.7)	59.4 (52-68.3)	62.5 (55.0-71.4)	64.4 (56.9-73.3)	66.7 (59.1-75.5)
Absolute RRR overestimate (%)	15.7 (6.7-25.8)	15.7 (6.8-25.8)	15.5 (6.7-25.4)	15.0 (6.4-24.7)	14.5 (6.1-24.0)	13.8 (5.7-23.0)	12.9 (5.2-21.7)	12.0 (4.7-20.7)	11.1 (4.2-19.2)
Observed RRR / True RRR	1.47 (1.14-2.12)	1.45 (1.14-2.04)	1.41 (1.14-1.94)	1.38 (1.13-1.84)	1.34 (1.11-1.75)	1.31 (1.10-1.66)	1.26 (1.09-1.56)	1.23 (1.08-1.50)	1.20 (1.06-1.43)

Data are median (IQR), or number (%)
